# Identification of the genetic mechanism that associates *L3MBTL3* to multiple sclerosis

**DOI:** 10.1093/hmg/ddac009

**Published:** 2022-01-28

**Authors:** Antonio Alcina, Maria Fedetz, Isabel Vidal-Cobo, Eduardo Andrés-León, Maria-Isabel García-Sánchez, Alicia Barroso-del-Jesus, Sara Eichau, Elia Gil-Varea, Luisa-Maria Villar, Albert Saiz, Laura Leyva, Koen Vandenbroeck, David Otaegui, Guillermo Izquierdo, Manuel Comabella, Elena Urcelay, Fuencisla Matesanz

**Affiliations:** Department of Cell Biology and Immunology, Instituto de Parasitología y Biomedicina "López Neyra", Consejo Superior de Investigaciones Científicas (IPBLN-CSIC) 18016 Granada, Spain; Department of Cell Biology and Immunology, Instituto de Parasitología y Biomedicina "López Neyra", Consejo Superior de Investigaciones Científicas (IPBLN-CSIC) 18016 Granada, Spain; Department of Cell Biology and Immunology, Instituto de Parasitología y Biomedicina "López Neyra", Consejo Superior de Investigaciones Científicas (IPBLN-CSIC) 18016 Granada, Spain; Bioinformatic Unit, Instituto de Parasitología y Biomedicina López Neyra (IPBLN-CSIC), 18016 Granada, Spain; UGC Neurología, Nodo Hospital Universitario Virgen Macarena, Biobanco del Sistema Sanitario Público de Andalucía, 41009 Sevilla, Spain; Genomics Unit, Instituto de Parasitología y Biomedicina López Neyra (IPBLN-CSIC), 18016 Granada, Spain; UGC Neurología, Hospital Universitario Virgen Macarena, 41009 Sevilla, Spain; Servei de Neurologia-Neuroimmunologia, Centre d'Esclerosi Múltiple de Catalunya (Cemcat), Institut de Recerca Vall d'Hebron (VHIR), Hospital Universitari Vall d'Hebron, Universitat Autònoma de Barcelona, Barcelona 08035, Spain; Departments of Immunology, Hospital Ramon y Cajal, (IRYCIS), 28034 Madrid, Spain; Servicio de Neurología, Hospital Clinic and Institut d'Investigació Biomèdica Pi i Sunyer (IDIBAPS), Institut de Neurociències, Universitat de Barcelona, 08036 Barcelona, Spain; Instituto de Investigación Biomédica de Málaga-IBIMA, UGC Neurología, Hospital Regional Universitario de Málaga, Málaga 29010, Spain; Inflammation & Biomarkers Group, Biocruces Bizkaia Health Research Institute, Barakaldo 48903, Spain; IKERBASQUE, Basque Foundation for Science Bilbao 48013, Spain; Neurosciences Area, Biodonostia Health Research Institute, 20014 San Sebastián, Spain; Multiple Sclerosis Unit, Neurology Service, Vithas Nisa Hospital, Seville 41950, Spain; Servei de Neurologia-Neuroimmunologia, Centre d'Esclerosi Múltiple de Catalunya (Cemcat), Institut de Recerca Vall d'Hebron (VHIR), Hospital Universitari Vall d'Hebron, Universitat Autònoma de Barcelona, Barcelona 08035, Spain; Lab. of Genetics of Complex Diseases, Hospital Clinico San Carlos, Instituto de Investigacion Sanitaria San Carlos (IdISSC), Madrid 28040, Spain; Department of Cell Biology and Immunology, Instituto de Parasitología y Biomedicina "López Neyra", Consejo Superior de Investigaciones Científicas (IPBLN-CSIC) 18016 Granada, Spain

## Abstract

Multiple sclerosis (MS) is a complex and demyelinating disease of the central nervous system. One of the challenges of the post-genome-wide association studies (GWAS) era is to understand the molecular basis of statistical associations to reveal gene networks and potential therapeutic targets. The L3MBTL3 locus has been associated with MS risk by GWAS. To identify the causal variant of the locus, we performed fine mapping in a cohort of 3440 MS patients and 1688 healthy controls. The variant that best explained the association was rs6569648 (*P* = 4.13E-10, odds ratio = 0.71, 95% confidence interval (CI) = 0.64–0.79), which tagged rs7740107, located in intron 7 of L3MBTL3. The rs7740107 (A/T) variant has been reported to be the best expression and splice quantitative trait locus (eQTL and sQTL) of the region in up to 35 human genotype-tissue expression (GTEx) tissues. By sequencing RNA from blood of 17 MS patients and quantification by digital qPCR, we determined that this eQTL/sQTL originated from the expression of a novel short transcript starting in intron 7 near rs7740107. The short transcript was translated into three proteins starting at different translation initiation codons. These N-terminal truncated proteins lacked the region where L3MBTL3 interacts with the transcriptional regulator Recombination Signal Binding Protein for Immunoglobulin Kappa J Region which, in turn, regulates the Notch signalling pathway. Our data and other functional studies suggest that the genetic mechanism underlying the MS association of rs7740107 affects not only the expression of L3MBTL3 isoforms, but might also involve the Notch signalling pathway.

## Introduction

Multiple sclerosis (MS) is a common inflammatory and demyelinating disease of the central nervous system. Its aetiology is not fully understood, but evidence exists supporting the involvement of environmental and genetic factors that trigger the disease (1). In the last 15 years, several genome-wide association studies (GWAS) have contributed to a better understanding of these genetic factors involved in the disease (2). In addition to HLA, the first locus associated with the disease and the one displaying the largest effect, over two hundred risk loci have been identified (3).

Nowadays, the definition of functional alterations underlying these genetic associations is key objective and faces great difficulties. Most of the variants associated with MS are not located in coding regions and their effects must be sought in the alteration of transcriptional regulation, which opens the possibility of influencing a large number of genes. Expression quantitative trait loci (eQTL) and splicing quantitative trait loci (sQTL) are powerful tools to identify causal genes and functional effects in MS-associated variants and other diseases (4–8). They link trait-associated genetic variants to functional effects at the transcriptional level. International projects such as the genotype-tissue expression (GTEx) have built eQTL databases to describe the relationship between genetic variation and gene expression in human tissues (9). Although these databases are very useful, important questions remain to be solved regarding how each variant exerts its effect on the RNA levels of affected transcripts and how they are related to the disease.

The association of the rs4364506 variant, located at the intron 12 of the *L3MBTL3* gene, with MS susceptibility was identified in a GWAS by Andlauer et al. (10). The association of this locus was corroborated by the last meta-analysis performed by the International Multiple Sclerosis Genetic Consortium, which identified 233 statistically independent associations with MS susceptibility using data from 47 429 MS cases and 68 374 controls (3). To date, no studies have established whether the MS-associated variant in the locus is functionally altering the *L3MBTL3* gene or another gene in the vicinity.

In the present study, we performed a fine-mapping of the region to define the causal variant responsible for the association with MS susceptibility, and to identify the effect of this variant on the splicing, transcription and expression of the *L3MBTL3* gene. We could correlate the causal variant with a new transcription initiation site within intron 7, that originates a previously undescribed transcript, which codified a truncated protein lacking the N-terminal region, involved in interacting with other proteins such as the transcriptional regulator Recombination Signal Binding Protein for Immunoglobulin Kappa J Region (RBPJ) that influences in the Notch signalling pathway (11).

## Results

### Identification of the causal variant by a tag-SNP approach

First, we addressed the validation of the association with MS of the *L3MBTL3* locus by genotyping the rs4364506 variant (10) in a Caucasian cohort of 3440 MS patients and 1688 healthy controls from Spain. The cohort was described in Gil-Varea et al. (12). Our results corroborated the association of the variant with MS [*P* = 4.80E-5, odds ratio (OR) = 0.81 (0.73–0.9].

Second, we performed fine-mapping of the region on chromosome 6 with coordinates from 129949823 to 130 205 324 (hg38). SNPs were selected by pairwise tagging of the EUR population from The 1000 Genome Project (13). The selection criteria captured all variants in the region with linkage disequilibrium *r*^2^ > 0.4 with the rs4364506 variant, initially associated with MS by GWAS. We selected 6 SNPs that captured 86 markers with *r*^2^ ≥ 0.7 (mean *r*^2^ = 0.94) and a minor allele frequency ≥0.1 (Supplementary Material, Table S1 and Supplementary Material, Fig. S1).

The six tag-SNPs were genotyped in the same cohort used to replicate the rs4364506 association. Genotype frequencies were fitted to an additive model. The best-associated SNP was rs6569648 [*P*-value = 4.13E-10, OR = 0.71 (0.64–0.79)] (Table 1). We performed a logistic regression analysis to establish the effect of other independent variants, testing the addition of each SNP to rs6569648, and adding this polymorphism to each one of the other tag-SNPs. The results showed that, in addition to generating the strongest signal, rs6569648 alone was sufficient to model the association of the locus.

**Table 1:** MS-association of the Tag-SNPs in the *L3MBTL3 locus*

SNP	Position Chr 6 (hg38)	Allele effect	*P*-value	OR (95% CI)	*P* effect conditional
rs6569648	130 027 974	C	4.13E-10	0.7143 (0.64–0.79)	NA
rs9388768	130 052 957	C	0.0007179	0.8531 (0.78–0.94)	0.43
rs4364506	130 068 795	A	4.80E-05	0.8123 (0.73–0.90)	0.51
rs6569650	130 082 671	A	0.0001449	0.8421 (0.77–0.92)	0.36
rs13211683	130 096 688	C	2.98E-05	0.8147 (0.74–0.90)	0.44
rs7740188	130 024 960	G	0.001014	0.8525 (0.78–0.94)	0.06

OR, odds ratio; CI, confidence interval.

### The MS-associated variant is an eQTL and sQTL for the *L3MBTL3* gene

To ascertain whether rs6569648 could correlate with the expression of any gene in the region, we explored the GTEx database (9). We observed that this variant is an eQTL for the *L3MBTL3* gene in 35 human tissues, showing the best correlation in spleen [normalized effect size (NES) = −1.1; *P*-value =1.7 × 10^−41^]. We also observed that this variant is a sQTL for the *L3MBTL3* gene in 26 tissues, showing the highest correlation in pituitary (NES = 1.4; *P*-value =10^−42^) and spleen (NES = 1.3; *P*-value = 9.3 × 10^−34^).

**Figure 1:**
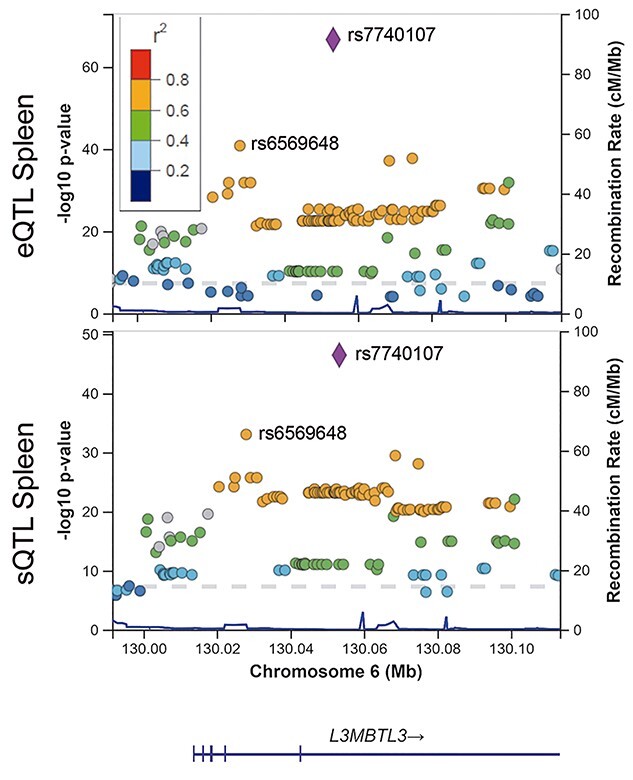
The best MS-associated variant rs6569648 is in high LD (*r*^2^ = 0.79) with the best eQTLs and sQTLs for *L3MBTL3* gene as shown in the LocusZoom plot from spleen GTEx data. Colours refers to the LD with rs7740107 variant using the European population of The 1000 Genomes project as reference, coordinates are in hg38.

To evaluate whether rs6569648 was the causal variant driving these expression effects, we scrutinized the GTEx results and found that the best eQTL and sQTL was rs7740107 in all tissues analysed. This variant was tagged by rs6569648 in the fine-mapping study (Supplementary Material, Table S1). The LD between these two variants was *r*^2^ = 0.79 and D′ = 0.99 for the European population. Interestingly, the pattern of variants associated with *L3MBTL3* expression and altered splicing was almost identical (Fig. 1), which led us to think that both effects were related.

### The *L3MBTL3* locus has also been associated with other traits and diseases

In the present work, we analysed other traits that have been reported associated with this locus. Sixteen associations with traits such as white blood cell count, monocyte count, lymphocyte count, height, hair colour, body mass index, breast cancer, or hip circumference have been published for the rs6569648 variant in 11 well-powered GWAS studies (14–24).

Eleven associations of rs7740107 have been described with immature reticulocyte fraction, red blood cell count, appendicular lean mass, estimated glomerular filtration rate, chronic kidney disease, white blood cell count, height, body mass index and haematocrit (14,15,17,21,25–28). Other variants, in LD with those reported here and associated with MS at the locus, have been also associated with other traits or diseases (Supplementary Material, Fig. S2), most of them related to blood cell counts and biometric features.

### rs7740107 is associated with transcription arrest and a new transcription initiation at *L3MBTL3* intron 7

**Figure 2:**
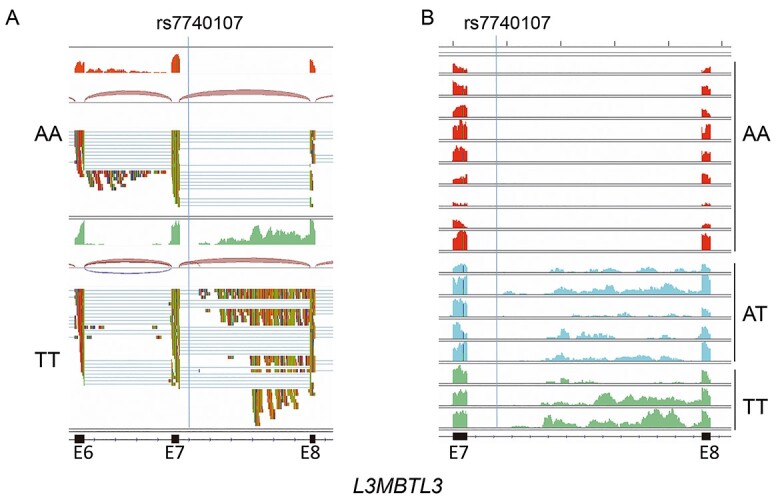
Transcription initiation at intron 7 of *L3MBTL3*. **(A**) RNA-Seq data from blood of two MS patients carrying TT or AA genotypes for the rs7740107 variant, represented by Integrative Genomics Viewer (IGV). The region depicted corresponds to chr6:130,051,116–130,055,425 (hg19), showing exons (E) 6, 7 and 8. From bottom to top, for each sample, are represented: alignment track, joints track and reads coverage track. (**B**) Reads coverage track from RNA-Seq data of 17 MS patients grouped by rs7740107 genotypes.

Since the sQTL and eQTL of the locus appeared to be related (Fig. 1), we further examined the region in which GTEx described the sQTL, defined as the difference in intron-excision ratio among genotypes. This region is located at position 130 052 991:130055171 (hg 38) on chromosome 6, overlapping intron 7 of the *L3MBTL3* gene. Interestingly, rs7740107 was also located in intron 7, at 325 bp of the splice donor. The RNA-Seq from two MS patient blood samples, carriers of TT or AA genotypes of the rs7740107 variant, showed multiple reads aligned to intron 7 in the TT but not in the AA sample (Fig. 2A). However, no new joints could be observed between these aligned sequences and the exons, ruling out novel alternative splicing. To confirm these results, the number of MS patients analysed was expanded to 17 carriers of TT, AT or AA genotypes for the rs7740107 variant (Fig. 2B). Reads in intron 7 were not observed in any of the 9 AA samples, whereas they were observed in the samples of the AT and TT carriers. These data proved that there were transcript initiations in intron 7 of the *L3MBTL3* gene with the T allele, the minor allele associated with MS protection.

**Figure 3:**
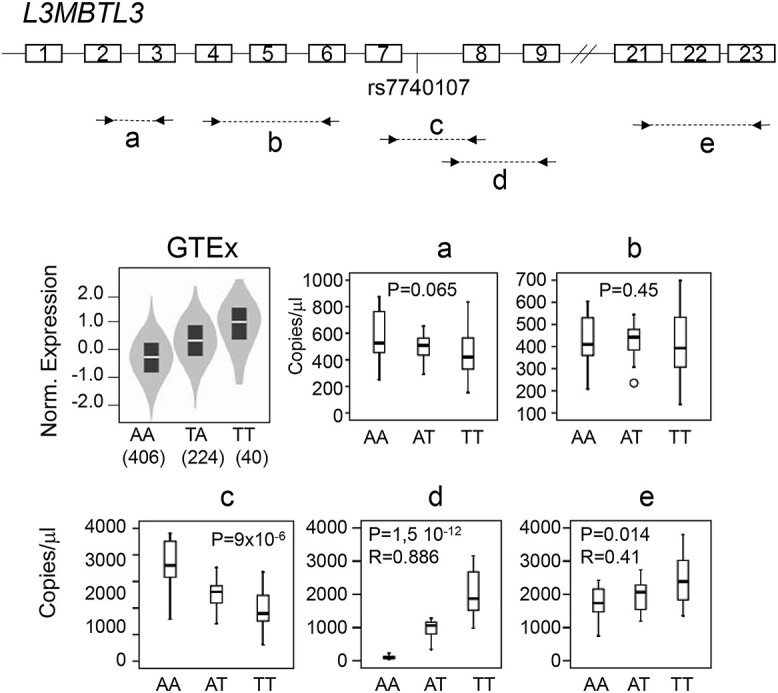
A novel transcript originating from intron 7 of *L3MBTL3* occurs in carriers of the rs7740107 T allele. (Top) Schematic of the *L3MBTL3* gene showing the location of the primers used for reverse transcriptase and quantification by digital qPCR. The exon number and position of rs7740107 are indicated. (Bottom) The graphs represent *L3MBTL3* expression levels with respect to rs7740107 genotypes: (GTEx) violin plot representing RNA-Seq of blood cells from GTEx; (**a**) to (**e**) box plots representing quantification by RT-digital qPCR of *L3MBTL3* expression levels using the different primers depicted at the top. RNA samples were purified from blood of 36 individuals: 14 AA, 11 AT and 11 TT carriers.

The eQTL for rs7740107 described by GTEx shows that *L3MBTL3* mRNA levels were higher for TT carriers in whole blood cells (Fig. 3), as well as in other tissues. To validate eQTL in blood samples from MS patients, and also to examine whether the transcription initiation observed from RNA-Seq data affects gene expression, we designed PCR primers at different positions of the gene and performed reverse transcription (RT) and digital qPCR in samples from 36 individuals: 14 AA, 11 AT and 11 TT genotype carriers. We did not detect significant differences in expression levels with amplification primers localized between exon 2 and exon 3 or exon 4 and exon 6 (Fig. 3a and b). However, we detected lower expression in TT carriers when we amplified between exon 7 and 8, flanking the intron where transcription initiation was observed (Fig. 3c). Opposite results were observed when we quantified *L3MBTL3* expression between intron 7 and exon 9 (Fig. 3d). The forward primer was located in intron 7, 35 bp upstream of the exon 8 acceptor splice site. These primers could only amplify transcripts originating from the intron, and not those with intron 7 spliced. Higher expression was also observed with primers located between exon 21 and exon 23 in the AT and TT samples compared with the AA samples (Fig. 3e). However, in the latter amplification, we cannot distinguish between transcripts originating from the 5′ end of the gene or from intron 7.

### Translation of *L3MBTL3* transcripts into proteins

**Figure 4:**
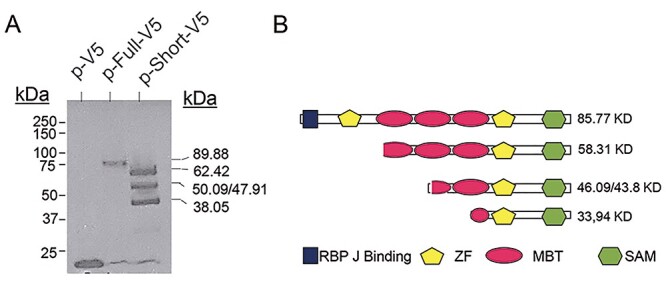
Expression of recombinant *L3MBTL3* full-length and short transcripts obtained from blood of a patient bearing rs7740107 TT alleles. (**A**) Western blot of extracts from HEK293T cells transfected with constructs carrying the full-length (p-Full-V5) and short (p-Short-V5) cDNA inserts, cloned into pcDNA™6.2/V5 directional expression plasmid (p-V5). The proteins were detected with an anti-V5 antibody. Molecular weight markers are at the left of the blot. Expected molecular weight of the full-length and short protein-V5 bands are indicated at the right side of the blot. (**B**) Scheme of the L3MBTL3 protein (XP_006715641.1) with the functional domains as described by Xu et al. (11): binding site for RBPJ (recombination signal binding protein for immunoglobulin kappa J region, blue); two ZF (C2C2 zinc finger, yellow); three MBT (malignant brain tumour domain, red); one SAM (sterile alpha motif, green). The expected molecular weight of the produced proteins, according with the possible starting ATG translation sites are indicated.

To demonstrate whether the transcript originating from intron 7 (short transcript) can be translated into protein, we cloned both the full-length and short transcripts obtained from a carrier of the TT genotype (Fig. 4). For cloning, the forward primer was placed in the 5’ UTR region in exon 2 for the full-length transcript, and at the end of intron 7 for the short transcript. The reverse primer was placed in the last 20 nt of the coding mRNA of exon 23. Both cDNAs were cloned into the plasmid pcDNA6.2/V5/GW/D-TOPO to produce the L3MBTL3 proteins fused to the V5 peptide at the carboxyl terminus. The plasmids were transfected into HEK293T cells and protein production was detected by western blot using an anti-V5 antibody. As expected, cells transfected with the full-length isoform produced the complete protein fused to the V5 fragment. The short isoform originated 3 protein bands with sizes compatible with initiation at the first, second or third, and fourth AUG translation star codon, respectively (Fig. 4). This was also supported by sequence analysis around the translation initiation codons looking for the presence of Kozak consensus sequences (data not shown) (29).

## Discussion

In the present study, we searched for the causal variant of the MS-associated *L3MBTL3* locus found by Andlauer et al. (10) in a GWAS. Our main finding revealed that in a fine-mapping of 3450 MS patients and 1688 healthy controls, the variant that best explained the association with MS was rs6569648. This polymorphism is a proxy for rs7740107, located in intron 7 of the *L3MBTL3* gene. We showed that this variant correlates with decreased expression of the full-length transcript of the *L3MBTL3* gene and with the initiation, in intron 7, of a new shortened isoform, which translates into three N-terminal truncated proteins.

Our results were in apparent contradiction with the eQTL described by GTEx, as they showed higher expression of *L3MBTL3* in TT carriers, whereas we observed differences in expression levels depending on the primers used for RT-digital qPCR quantification. These differences are due to the fact that GTEx performs expression quantification at the gene level, using an isoform collapsing procedure to a single transcript model for each gene. In our case, the primers were designed to capture the expression of the different isoforms, which allowed us to detect the new transcript originating from intron 7. This transcript has not been previously described in Ensembl or GENCODE databases. The GTEx results would have led us to conclude that the causal effect associated with MS protection would be the increased expression of *L3MBTL3*. Although the number of RNA-Seq reads mapped in the *L3MBTL3* gene is higher in T allele compared with A allele carrier, they are distributed in different transcripts. In addition to the eQTL, GTEx also described a sQTL in intron 7. They used LeafCutter (30), a system based on finding introns that share splice sites to detect split reads and uncover alternative intron-excision options. The sQTL from GTEx in intron 7 allowed us to design RT-digital qPCR assays around the variant to reveal the effect on *L3MBTL3* transcription. Our data suggested that the functional effect underlying the MS-association might lie in the reduction of full-length protein expression, and/or in the appearance of N-terminal truncated proteins that might have distinct activity.

As our data suggest, the truncated L3MBTL3 proteins lost the amino-terminal region, which carries several functional domains. L3MBTL3 is a regulator of Notch: down-regulation of L3MBTL3 leads to up-regulation of Notch target genes, while its overexpression produces the opposite effect, i.e. repression of gene expression (11). This balanced regulation is mediated by RBPJ, which interacts with amino acids 1–64 in the N-terminus of L3MBTL3. In addition, the truncated protein has a dominant effect over the full-length protein. It is tentative to speculate that the alteration of Notch signalling in an opposite manner between the full-length and truncated proteins could be the functional reason of the locus association with MS. Notch has an important role in adult neurogenesis and oligodendrocyte differentiation, and it is also essential for T cell development (31–33).

On the other hand, the truncated proteins translated from the short transcript also lack malignant brain tumour (MBT) interaction domains. It has been demonstrated that the L3MBTL3 MBT domains can directly recognize SOX-2 methylated Lys-42 (34) and also the K142-methylated DNMT1 (35) proteins and regulates their degradation. This open de possibility that the cells of individuals carrying rs7740107 T allele could have altered the homeostasis of SOX2 and DNMT1 proteins. SOX2, is a master transcriptional factor involved in the regulation of embryonic development, determination of cell fate and maintenance of stem-cells in the central nervous system (36,37). Moreover, DNMT1 is a major DNA methyltransferase that methylates cytosine residues in the CpG dinucleotides of the genome and is he major enzyme responsible for maintaining methylation patterns following DNA replication to preserve epigenetic inheritance (38).

As mentioned, several other traits or diseases are associated with the same polymorphism or are in very high LD with the MS variant. Among these traits, no other autoimmune diseases were found, as commonly happens in other MS-risk polymorphisms (39). These traits do not seem to have much in common, but the possible effect of the variant on Notch signalling pathway, maintenance of CpG DNA methylation patterns by DNMT1 or regulation of the self-renewal of stem cells by SOX2 are mechanisms key on the growth, development and differentiation of multiples tissues, which could explain the implication of the genetic variant in so different traits.

This work evidences the complexity of transcending the original GWAS signals towards the identification of the actual etiologic polymorphisms, the genes involved and their products and/or regulatory roles. Even though the genomic approaches open a valuable panorama, a close look also helps in their correct interpretation.

## Material and Methods

### MS patients and healthy donors

Validation and fine mapping of the *L3MBTL3* locus was performed with DNA samples from a cohort of 3450 MS patients and 1688 age- and sex-matched healthy donors of European origin, recruited from 8 Spanish MS centres. The demographic characteristics of the cohort are described in Gil-Varea et al. (12). RNA-Seq was performed on blood from 16 MS donors and 1 healthy donor. Of the MS donors, 9 had relapsing–remitting MS (RRMS) and 9 secondary progressive MS. None of the donors had received any treatment in the 3 months prior to blood collection. For RT-digital qPCR, in order to have balance number of samples of each genotype and since the frequency of TT genotype is around 6% on Caucasian population, we preselected 14 AA, 11AT and 11TT carriers out of 100 patients and 100 healthy controls. Out of the 32 samples, 12 samples were from MS patients and 20 from healthy controls. There was not significate L3MBTL3 expression difference associated within gender or disease, therefore we performed the eQTL calculation using data from MS and healthy controls combined. In Supplementary Material, Table S3, we show the demographic characteristics of the cohort used for expression analysis. All participants signed the informed consent form and the study was approved by the Andalusian Ethics Committee.

### SNPs genotyping

Genotyping of rs4364506 was performed using iPLEX® Gold chemistry at the National Genotyping Center (CeGen-PRB2, USC node). The remaining SNPs genotyped for fine mapping were performed in the same patient and healthy donor cohorts using pre-designed TaqMan assays by Thermo Fisher Scientific (Thermo Scientific, IL, USA). The assays are listed in Supplementary Material, Table S2. Random samples were genotyped in duplicate to check for reproducibility. Genotyping data were analysed with PLINK V.1.07 (40). Logistic regression models were calculated to determine the independence of effects. Genotypes of rs7740107 from blood donor were obtained from GWAS data performed with Infinium Global Screening Array, GSA (Illumina, CA, USA). Deviation from Hardy–Weinberg equilibrium for all SNPs was tested by exact test.

### RNA-Seq from blood

The RNA-Seq data were obtained from seventeen RNA samples extracted from stabilized whole blood using the PAXgene Blood RNA Kit (QIAGEN GmbH, Hilden, Germany) and quantified using Nanodrop One® and Qubit® (Thermo Scientific, IL, USA). Library preparation and Illumina sequencing were carried out at the IPBLN Genomics Facility (CSIC, Granada, Spain). Total RNA quality was verified by Bioanalyzer RNA 6000 Nano chip electrophoresis (Agilent Technologies, CA, USA). All RNA samples showed a RIN value above 8. In order to increase detection sensitivity, the alpha and beta globin mRNA was depleted using hybridization technology. Briefly, for each sample, 400 ng of total RNA were treated with the GLOBINclear™-Human Kit (Thermo Scientific, IL, USA), with a globin mRNA depletion efficiency>95% (tested by qPCR in a representative set of samples). The totality of recovered RNA was used as input for RNA-seq library construction performed with Truseq® stranded mRNA kit (Illumina, CA, USA) and IDT for Illumina® Truseq® UD RNA indexes kit. Quality and size distribution of PCR-enriched libraries was validated through Bioanalyzer High-Sensitivity DNA assay and the concentration was measured on the Qubit® fluorometer. Final libraries were pooled in an equimolecular manner, and then diluted and denatured as recommended by Illumina NextSeq 500 library preparation guide. The 75 × 2 nt paired-end sequencing was conducted on a NextSeq 500 sequencer, in two consecutive runs, using the high output configuration and producing 41.591.000 raw paired reads sample on average.

### RNA-Seq data analysis

To analyse transcriptomic samples, miARma-Seq pipeline was used (41). This workflow performs all steps from raw data to differential expressed genes (DEG) calculation. Firstly, raw data were evaluated using FastQC software to examine the quality of the reads (http://www.bioinformatics.babraham.ac.uk/projects/fastqc). No adapter accumulation or bad quality reads (q < 30) were found. In the second step, miARma-Seq aligns all sequences using HISAT2 (42), resulting in a 95.98% of properly aligned reads. With this aim, *Homo sapiens* Gencode v26 genome-build: GRCh37 was used as reference genome. Data were deposited at BioProject database with accession number PRJNA732389.

### RNA quantification by digital qPCR

To quantitate the expression of the *L3MBTL3* gene by RT-digital qPCR, 1500 ng of RNA were reverse transcribed into cDNA with oligodT and random primers, according to Superscript IV First-Strand Synthesis SuperMix protocol (Thermo Scientific, IL, USA). We performed digital qPCR in duplicates using QX200 Droplet Digital PCR EvaGreen Supermix (BioRad, USA). Data was analysed with QuantaSoft (BioRad, CA USA), which counts the fluorescent positive and negative droplets to calculate target DNA concentration. Primers for digital qPCR amplification were designed with Primer 3 V1.07 software, and their sequences are shown in Supplementary Material, Table S2.

### Amplification of the *L3MBTL3* full and short isoforms

Blood cells cDNA from a carrier of rs7740107*TT was amplified by PCR using the Q5® High-Fidelity DNA Polymerase (New England BioLab, MA, USA). The full isoform of *L3MBTL3* was amplified using a forward primer located in the 5′ UTR, position chr6.130022261–130 022 280 hg38. The short *L3MBTL3* isoform was amplified using a forward primer located in intron 7 of the *L3MBTL3* gene at position chr6:130055117–130 055 136. These two primers have 4 nt with sequence 5′-CACC for directional insertion into the plasmid. For both isoforms, the same reverse primer located at the last 20 coding nucleotides of the gene, position chr6:130012271–130 032 270, was used. PCR conditions were: 1 cycle of 94°C for 3 min; 34 cycles of 94°C for 30 s; 60°C for 30 s; 72°C for 2 min.

### Cloning and expression of the *L3MBTL3* isoforms in HEK293T

PCR products were cloned into the expression plasmid pcDNA6.2/V5/GW/D-TOPO (Thermo Scientific, IL, USA). Positive colonies were validated by Sanger sequencing. The full isoform cloned corresponded to the ENST00000533560 transcript, and the short isoform to the sequence of 35 bp of intron 7 near the splice donor plus exons 8 to 23. The recombinant plasmids for transfection were purified with the EndoFree Plasmid Maxi Kit (QIAGEN GmbH, Hilden, Germany). The HEK293T (human embryonic kidney [epithelial]) cell line was cultured in DMEM high glucose medium supplemented with 10% foetal calf serum and 100 U/ml penicillin/streptomycin (all from Gibco, Thermo Scientific, IL, USA). Following the manufacturer’s instructions, 2 × 10^5^ cells/well were seeded in a six-well plate and transfected the day after with the different recombinant plasmids and the control plasmid pcDNA™6.2/V5/GW-CAT using LTX lipofectamine reagent (Thermo Scientific, IL, USA). After 24 h of culture, cell extracts were dissolved in Laemmli buffer and processed by sodium dodecylsulfate polyacrylamide gel electrophoresis (SDS-PAGE) at 10% concentration and transferred to PVDF-P transfer membranes (Thermo Scientific, IL, USA). The blot membrane was incubated with an antibody against the V5 label (ab27671, Abcam, Cambridge, United Kingdom) and an HRP-conjugated mouse anti-IgG secondary antibody (H + L) (#62–6520, ThermoScientific, IL, USA). Protein bands were revealed with Pierce ECL Western Blotting substrate (Thermo Scientific, IL, USA). The molecular weight marker used as a guide was Precision Plus Protein™ All Blue Prestained Protein Standards (1 610 373, Bio-Rad, CA, USA).

## Supplementary Material

HMG-2021-CE-00558_Alcina_supplementary_material_ddac009
