# Supplementary material for: Identification of the genetic mechanism that associates L3MBTL3 to multiple sclerosis
Source: Hum Mol Genet. 2022 Jan 28;31(13):2155–63. doi: 10.1093/hmg/ddac009 (PMC9262392; doi:10.1093/hmg/ddac009)
Supplement: HMG-2021-CE-00558_Alcina_supplementary_material_ddac009 [file hmg-2021-ce-00558_alcina_supplementary_material_ddac009.pdf]

Supplementary Table 1. Tagged SNPs of the *L3MBTL3* locus

Tag-SNP	SNP captured
rs6569648	rs7740107, rs6569648
rs9388768	rs7746589, rs9372954, rs9388769, rs9375701, rs4551191, rs7761063, rs4351283, rs9492442, rs4279458, rs4373379, rs9375699, rs9385532, rs12197047, rs6569651, rs7774174, rs9375703, rs6914670, rs9375702, rs11759018, rs9385533, rs7760760, rs12201492, rs11752200, rs6900473, rs4339481, rs6923819, rs9375698, rs7763108, rs4594967, rs4321845, rs9375700, rs12661232, rs7451021, rs9402214, rs9388768, rs9402213, rs9388770, rs4404788
rs4364506	rs12190724, rs7762724, rs7769599, rs9483085, rs9492441, rs4548027, rs9492445, rs9483083, rs12198331, rs9483079, rs9492447, rs10499172, rs9492443, rs9483082, rs7759381, rs7764762, rs9492446, rs9492451, rs4364506, rs9492440, rs13209890, rs12206651
rs6569650	rs9321207, rs4897364, rs7756733, rs6569650, rs4321846, rs9398939
rs13211683	rs7756339, rs9492452, rs7755589, rs9492454, rs9492449, rs13211683
rs7740188	rs12661188, rs9388766, rs7744830, rs1415700, rs6926186, rs9388767, rs6899976, rs9375694, rs7740188, rs9375695, rs7756870, rs9402211

SNPs were selected by pairwise tagging from EUR population of *The 1000 Genome project*. The selected region was chr 6_129949823-130205324 from hg38. The 6 Tag-SNPs captured 86 markers with $r^2 \geq 0.7$ (mean $r^2 = 0.946$) and a minor allele frequency (MAF) ≥0.1

Supplementary Table 2. Oligonucleotide primers used in this study.

**ddPCR primers**	
a-Forward	GAGAGTGGACAGCAATTGTGAA
a-Reverse	GTAACGTGCCCACTCCATCT
b-Forward	AGTTTGGAGCCCTGGAAGTT
b-Reverse	TGAAAGGCATCCCAGTCTTC
c-Forward	TCAGAAGGAAGAAAGGGACGTA
c-Reverse	CTTTTCCTCCGTGCTCTCTG
d-Forward	TCACTGGTGCCAATTTCTTG
d-Reverse	CACCACGCTTTCTTTCCTTT
e-Forward	ATGGAGCACAGACGAGGTGT
e-Reverse	TCTTCTCTGCAGCTTTGAACA
**Cloning**	
5'-Full-length isoform	CACCTTGTGAAGATCGCCAGAGAG
5'-Short-length isoform	CACCACTGGTGCCAATTTCTTGA
3'-Primer	AAGTTCATTGTGAGAATTCTTC
**Tag-SNPs**	**TaqMan (Thermo Fisher Scientific)**
rs7740188	C___1823591_20
rs6569648	C____303990_10
rs9388768	C__29894205_20
rs6569650	C__27442386_10
rs13211683	C___1783737_20

Supplementary Table 3. Demographical characteristics of patients and healthy controls included in the RNAseq and RT-digital qPCR

	**MS (RNAseq/ddqPCR)**	**Healthy controls (RNAseq/ddqPCR)**
N	29 (16/13)	23 (1/22)
**Gender**		
Males	10	9
Females	19	14
**Age (years, med (P25-P75))**	63 (51-68)	
**Age at disease onset (years, med (P25-P75))**	26(23/31)	
**MS type**		
RR	21	
SP	8	
**EDSS (med (P25-P75))**	3 (1.5-7)	
**Treatment at extraction**		
Non in the previous 3 months	20	
Glatiramer acetate	1	
Interferon beta	3	
Natalizumab	2	
Fingolimod	3	

med median, *P25* 25th percentile, *P75* 75th percentile, *EDSS* Expanded Disability Status Scale.

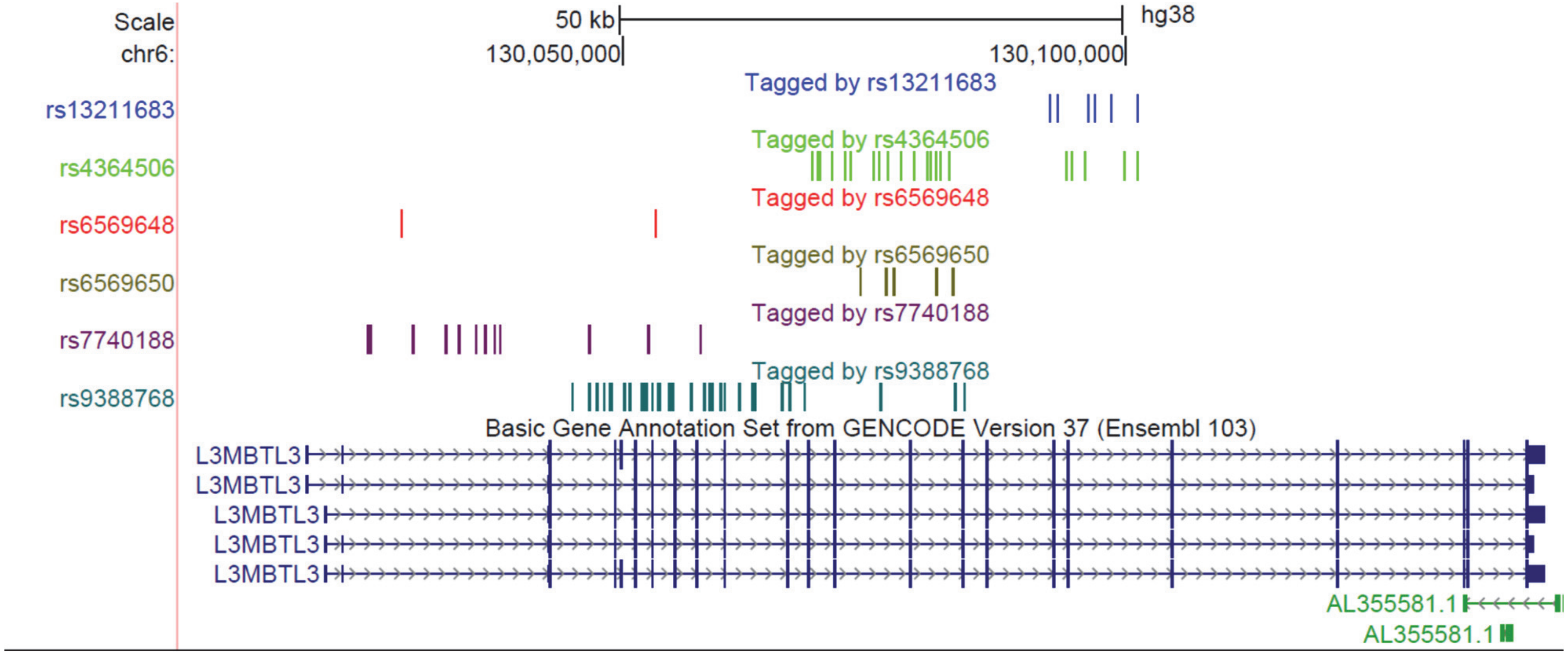

Supplementary Figure 1.
Location of the tagged SNPs at the *L3MBTL3* locus.

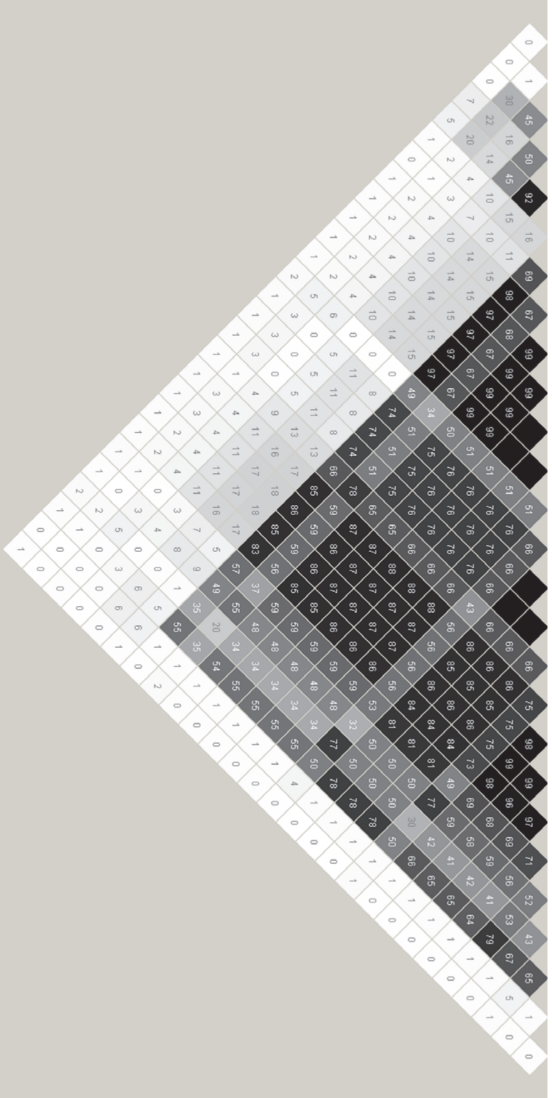

rs17757727	Heart rate in heart failure with reduced ejection fraction
rs6920244	Height
rs9492436	Highest math class taken (MTAG), Educational attainment (MTAG)
rs6569647	Height, Birth weight
rs113898003	Height, Heel bone mineral density
rs1415701	Height, Heel bone mineral density, Birth weight, Heel bone mineral density, Male-pattern baldness, Offspring birth weight,
rs7740188	Fat-free mass
rs6569648	Height, Body mass index, Lymphocyte counts, Breast cancer, Hip circumference,, Hair color, White cells and monocyte count
rs9402211	Waist-hip ratio
rs9388766	Waist-to-hip ratio adjusted for BMI, Hip circumference adjusted for BMI, C-reactive protein levels, Waist-hip ratio,
rs9375694	Estimated glomerular filtration rate, Triglyceride levels
rs6899976	Height, Heel bone mineral density
rs9375695	Heel bone mineral density
rs4897361	Lipid traits (pleiotropy) (HIPO component 2)
rs9385532	C-reactive protein levels
rs7746589	C-reactive protein levels
rs9388768	Hemoglobin levels
rs7740107	Immature fraction of reticulocytes, Red and white cell count, Height, Estimated glomerular filtration rate, Chronic kidney disease, Body mass index, Appendicular lean mass
rs11759018	Heel bone mineral density
rs12661188	Height, Hip circumference adjusted for BMI, Red blood cell count
rs7451021	Waist-to-hip ratio adjusted for BMI
rs9375702	Weight, Body mass index
rs12197047	Low density lipoprotein cholesterol levels, LDL cholesterol levels, Apolipoprotein B levels
rs4364506	Multiple sclerosis
rs4321846	Lung function (FEV1/FVC)
rs6569651	Post-bronchodilator FEV1 x air pollution (CO) interaction in childhood asthma
rs17633311	Height
rs61201007	PCKD

Supplementary Figure 2. Linkage disequilibrium (LD) plot of the SNPs of the *L3MBTL3* locus. Only the SNPs of the region that have been reported associated with a trait by GWAS are represented. Data obtained from GWAS catalogue (https://www.ebi.ac.uk/gwas/). LD in r^2 and the data used was EUR population from The 1000 Genomes project. Haploview (https://www.broadinstitute.org/haploview/haploview) was used to perform calculations and depict the figure. In red are indicated the SNPs studied in the present article.
